# Non-Invasive Retinal Vessel Analysis as a Predictor for Cardiovascular Disease

**DOI:** 10.3390/jpm14050501

**Published:** 2024-05-09

**Authors:** Raluca Eugenia Iorga, Damiana Costin, Răzvana Sorina Munteanu-Dănulescu, Elena Rezuș, Andreea Dana Moraru

**Affiliations:** 1 Department of Surgery II, Discipline of Ophthalmology, “Grigore T. Popa” University of Medicine and Pharmacy, Strada Universitatii No. 16, 700115 Iași, Romania; raluca-eugenia.iorga@umfiasi.ro (R.E.I.); andreea-dana.moraru@umfiasi.ro (A.D.M.); 2 Doctoral School, “Grigore T. Popa” University of Medicine and Pharmacy, 700115 Iași, Romania; 3 Department of Gastroenterology, “L. Pasteur” Clinical Hospital, 28630 Le Coudray, France; 4 Department of Internal Medicine II, Discipline of Reumathology, “Grigore T. Popa” University of Medicine and Pharmacy, 700115 Iași, Romania; elena.rezus@umfiasi.ro

**Keywords:** retinal vessel analysis, cardiovascular disease, retinal microvascular biomarkers, artificial intelligence

## Abstract

Cardiovascular disease (CVD) is the most frequent cause of death worldwide. The alterations in the microcirculation may predict the cardiovascular mortality. The retinal vasculature can be used as a model to study vascular alterations associated with cardiovascular disease. In order to quantify microvascular changes in a non-invasive way, fundus images can be taken and analysed. The central retinal arteriolar (CRAE), the venular (CRVE) diameter and the arteriolar-to-venular diameter ratio (AVR) can be used as biomarkers to predict the cardiovascular mortality. A narrower CRAE, wider CRVE and a lower AVR have been associated with increased cardiovascular events. Dynamic retinal vessel analysis (DRVA) allows the quantification of retinal changes using digital image sequences in response to visual stimulation with flicker light. This article is not just a review of the current literature, it also aims to discuss the methodological benefits and to identify research gaps. It highlights the potential use of microvascular biomarkers for screening and treatment monitoring of cardiovascular disease. Artificial intelligence (AI), such as Quantitative Analysis of Retinal vessel Topology and size (QUARTZ), and SIVA–deep learning system (SIVA-DLS), seems efficient in extracting information from fundus photographs and has the advantage of increasing diagnosis accuracy and improving patient care by complementing the role of physicians. Retinal vascular imaging using AI may help identify the cardiovascular risk, and is an important tool in primary cardiovascular disease prevention. Further research should explore the potential clinical application of retinal microvascular biomarkers, in order to assess systemic vascular health status, and to predict cardiovascular events.

## 1. Introduction

Cardiovascular disease (CVD) is the most common cause of death in the world. Cardiovascular mortality might be predicted by the alterations in the microcirculation, such as retinal vasculature [1]. The high mortality rate of coronary heart disease (CHD) highlights the necessity to detect it early. Current guidelines recommend approaches to identify individuals as high, intermediate, or low risk using risk prediction models such as age, gender, race, hypertension, diabetes, dyslipidaemia and cigarette smoking. Microvascular pathology plays an important role in the development of cardiovascular pathology [2].

The retinal vasculature is a unique biological model used to study microvascular abnormalities associated with CVD [3]. The retinal vasculature has also been described as “a window to the heart” [4]. This suggests that retinal parameters could potentially serve as biomarkers for cardiovascular disorders. In order to quantify microvascular changes in a non-invasive way, fundus images can be taken and analysed. The central retinal arteriolar (CRAE), the venular (CRVE) diameter and the arteriolar-to-venular diameter ratio (AVR) can be used as biomarkers to predict the cardiovascular mortality. This is the static retinal vessel analysis (SRVA) [5]. Narrower CRAE, wider CRVE and a lower AVR have been associated with an increased risk of coronary heart disease [6]. Moreover, narrower retinal arterioles have been associated with reduced myocardial perfusion, as detected by cardiac magnetic resonance imaging [7]. On the other hand, dynamic retinal vessel analysis (DRVA) allows the quantification of retinal changes using digital image sequences in response to visual stimulation with flicker light. Recent studies show the importance of SRVA and DRVA as screening tools for CV risk and disease detection.

It is important to identify alterations in the retinal vascular bed in order to better understand the manifestation of systemic cardiovascular disease. The study of retinal vasculature may help identify the subclinical microvascular alterations associated with cardiovascular disease [8].

**Figure 1:**
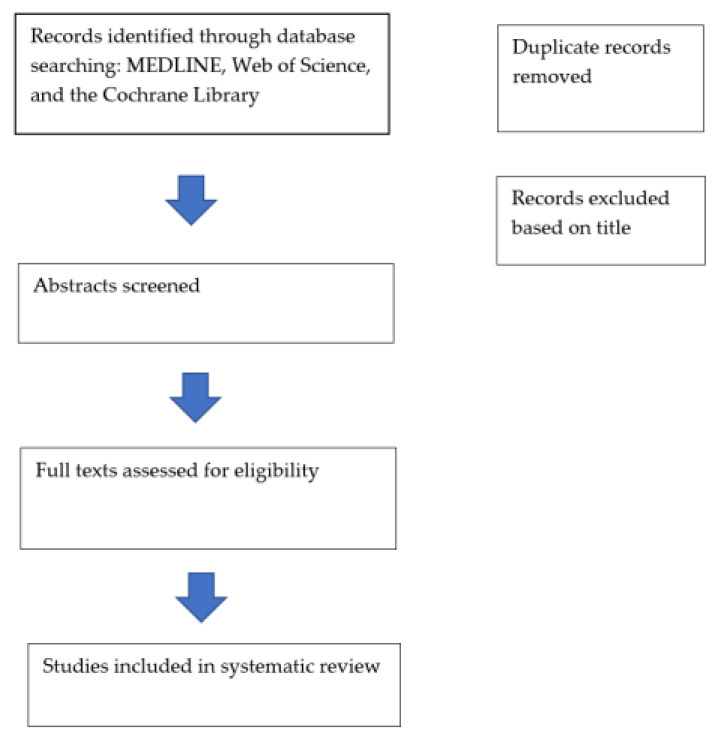
Flowchart.

This article is a review of previous studies and does not contain new studies with human participants. In our study, we performed a review of literature, using MEDLINE (PubMed), Web of Science (Clarivate Analytics), and the Cochrane Library (Cochrane) (Figure 1). We intended to emphasize the role of retinal vessel analysis—based on fundus photographs and OCT imaging—that can be used as a tool in the cardiovascular disease prevention and management. The main inclusion criteria were the quality of the research and the focus on retinal vascular imaging, oculomics, artificial intelligence and CVD. Our search was focused on studies published in the last 10 years. We used keywords from the medical field such as “retinal vessels”, “fundus photographs”, “optical coherence tomography–angiography”, “cardiovascular risk factors”, “cardiovascular pathologies”, which we combined with keywords from the machine learning field—“artificial intelligence”, “deep learning”.

## 2. Anatomy and Physiology of Retinal Vasculature

In the retinal vessels, the permeability is higher and the endothelium is more vulnerable to oxidative stress [9]. The retinal endothelium is affected in the presence of reactive oxygen species, as it contains less of the protective superoxide dismutase. ROSs are involved in the development of atherosclerosis and cardiovascular pathologies [10].

Retinal microcirculation is an end-arterial system that contains no anastomosis and no capillary sphincters. The vessels that form the retinal microcirculation are the small arteries, arterioles, capillaries, venules and small veins. The wall of small arteries and arterioles consists of a thick layer of vascular smooth muscle cells. The capillary bed links the terminal arterioles and venules. The walls of post-capillary venules and veins contain a thin layer of smooth muscle.

It is important to know that retinal vessels are up to 25% larger in the temporal quadrant than in the nasal retina. Even if the blood flow is 2–3 times larger in the temporal quadrant of the retina, the blood flow of the superior and inferior temporal quadrants does not differ [11]. On the other hand, Garfoher et al. used OCT measurements in order to report differences in blood flow between the superior and inferior temporal quadrants of the retina [12].

The retina is known to have the highest oxygen consumption per volume in the human body. Blood flow autoregulation is maintained by pressure autoregulation—adaptations of retinal arterioles to changes in perfusion pressure [13] and metabolic autoregulation [14]. High levels of O_2_ induce a decrease in retinal vessel diameters, and thus a decrease in blood flow, in order to prevent excessive oxygen exposure [14].

Using optical coherence tomography angiography (OCTA), Kalab et al. showed that flicker light induces an increase in retinal blood flow of about 40% in arteries and 30% in veins. They also noted an increase in microvascular density, more marked in the superficial capillary plexus [15]. Flicker light exposure activates neurons and astrocytes that release neurotransmitters. These neurotransmitters initiate a signalling cascade that induces dilation of retinal arterioles and venules, due to the vasoactive substances (NO, adenosine, prostaglandins) [16]. Blood velocity is increased in the large pre-capillary and post-capillary vessels of the retina, leading to flow-induced vasodilation of larger retinal vessels. This modification can be quantified by using DRVA.

## 3. Retinal Vessel Analysis

Retinal fundus colour imaging is a common procedure for evaluating vessel’s structure and it is used as a tool for early detection of various forms of retinopathy. The retinal vascular bed can also be examined by using optic coherence tomography angiography or adaptive optics imaging (Table 1).

### 3.1. Retinal Vessels Analyser Using Fundus Images

In recent years, retinal vessel identification studies have been attracting more attention due to non-invasive fundus imaging. There are different fundus cameras available that allow concomitant photographs taking and image analysis. Retinal vessel classification faces some challenges that make it difficult to obtain high accurate results. Currently, retinal vascular alterations are either manually or semi-automatedly assessed following standardized grading protocols. Most recently, artificial intelligence (AI), in particular deep learning (DL) with convolutional neural network (CNN), have been developed in ophthalmology, in order to facilitate image interpretation [18]. With the help of DL, some cardiovascular risk factors can be quantitatively predicted—age, gender, blood pressure, body mass index, smoking [19].

#### 3.1.1. Types of Software Used to Measure Retinal Vasculature

Many large epidemiological studies used digitized images to measure the retinal arteriolar and venular diameters [20,21]. Software such as the Retinal Analysis (RA) and Integrative Vessel Analysis (IVAN) was used to measure arteriolar and venular calibre in the retinal vasculature from digital photographs. The revised Knudtson–Parr–Hubbard formula summarizes the retinal arteriolar and venular calibres of six large arterioles and venules (CRVE) [22]. The arteriolar–venular ratio (AVR) was used as a marker for early detection of cardiovascular diseases. In the United States of America, in optic disc-centred images, investigators consider for ARV calculation the six largest vessels in the area within 0.5–1 optic disc diameter from its margins [23]. Vessels’ classification on retinal fundus images faces some challenges. The classification approaches are based on visualization of specific geometric features in the retinal vasculature bed, which discriminate arteries from veins. Normally, veins are thicker and darker than arteries, central reflex is easily seen in arteries, and arteries and veins usually alternate near the optic disc. These features are insufficient to distinguish these two types of retinal vessels.

Retinal vessel analysis on fundus images includes five stages: vessel segmentation, selection of the region of interest, feature extraction for each vessel, classification of the feature vectors and, in the end, a combination of the results for final vessel labelling [24].

Singapore I Vessel Assessment (SIVA) automatically detects the optic disc centre and the retinal arterioles and venules. It also detects additional geometry parameters (branching, bifurcation, tortuosity), and may detect early microvascular damage [25,26].

VAMPIRE is the vessel measurement platform for retinal images. It quantifies some retinal vessel properties, such as vessel width, vessel branching, and tortuosity [27].

Automated retinal vessel analysis based on fundus photographs is a non-invasive method that helps predict the cardiovascular risk. Thus, it may have some limitations and might be challenging obtaining high-accuracy results. A major issue in classification is that the absolute colour of blood in the vessels of the same subject varies between images [28]. Vessel thickness is not a reliable feature for classification because it is affected by vessel segmentation. Thus, it may be a challenge to differentiate between arteries and veins. Some methods simplified this problem by choosing only major vessels around the optic disc head.

#### 3.1.2. Retinal Vascular Changes Used in Studying CVD

Retinal vascular changes can be classified as qualitative and quantitative. Qualitative retinal vascular changes can be further classified into classic retinopathy signs—such as microaneurysm, retinal haemorrhages, hard exudates and cotton-wool spots, and retinal arteriolar wall signs—focal arteriolar narrowing, arteriovenous nicking [29] (Table 2). Quantitative retinal vasculature can be measured with computer software and standardized photographic protocols.

### 3.2. Optical Coherence Tomography—Angiography OCTA

OCTA is a non-invasive method that helps visualise the retinal vasculature. OCTA allows one to analyse different features of vascular pathologies, such as impaired vascular perfusion, neovascularization, cotton wool spots [30]. Also, some devices provide information on quantitative retinal vascular metrics, such as vessel density, vessel perfusion and flow index [31].

OCTA represents an alternative to fluorescein-angiography. It provides important data regarding the retinal vascular network—vessel density, vessel diameter index, the fractal dimension, branching angles [32,33]. Using specific algorithms, OCTA evaluates capillaries and large vessels separately [34,35]. A group of authors studied vessel density during the transition from light to darkness. They noticed an increase in vessel density in the superficial capillary plexus and a decrease in the intermediate and deep capillary plexus [36]. Vitreous floaters and eye movement can lead to artefacts [37]. In order to analyse retinal blood flow, it is important to determine the vessel diameter. The diameters of retinal vessels measured from OCTA were larger than those measured on fundus photographs [38]. The pixel resolution of OCTA images is approximately 3.85 to 4.14 mm per pixel. This suggests that alterations in retinal vascular diameter may not be detected by OCTA [39].

#### 3.2.1. Choroidal Vasculature Imaging

The choroid is a tissue with the highest vessel density in the body. OCT provides a non-invasive evaluation of the vascular status of a patient. Ahmad et al. revealed a thinner choroid in patients with coronary artery disease and heart failure than in healthy controls [40]. These findings help us correlate outer retinal health and systemic cardiovascular health.

#### 3.2.2. Imaging of the Retinal Capillary Network

OCTA helps us quantify the capillary network with the use of new image analysis methods, identifying microvascular abnormalities [41]. Takayama et al. suggested that OCTA might help evaluate the progression of arterial hypertension [42]. In his study on adults with systemic hypertension, Chua found correlations between OCTA features and cardiovascular risk factors [43]. All these findings highlight the importance of OCTA in early detection of microvascular changes at capillary level.

### 3.3. Reference Values of Retinal Microcirculation Parameters

Normative data for retinal vasculature was provided by the Gutenberg Health Study, by analysing fundus photographs from 4309 participants [44]. The authors determined the CRAE, the CRVE and the AVR. The mean values for CRAE, CRVE and AVR were 178.37 ± 17.91 µm, 212.30 ± 17.45 µm, and, respectively, 0.84 ± 0.07 µm. All these parameters were higher in women, when compared to men (Table 3).

Systemic hypertension was associated with lower AVR and CRAE values, being lower in participants with uncontrolled hypertension (172.28 µm, range 83.05–251.04; and 0.81 µm, range 0.56–1.04 [44] (Table 4).

Cifkova et al. analysed the retinal microcirculation using laser flowmetry. They found that the blood flow in the capillaries around the optic nerve head increased with age, while vessel and luminal diameters decreased. Systolic blood pressure correlated significantly with wall thickness. The authors also showed a positive relation between carotid femoral pulse wave velocity and wall thickness, indicating the close link between micro- and macro-vasculature [45].

The African-PREDICT study calculated CRAE and CRVE from retinal images, and determined the vessel calibre responses to flicker light induced provocation. They found that black participants had a smaller CRAE value (158 µm ± 11 vs. 164 µm ± 11) than their white counterparts. CRVE measurements were similar in the two groups. In response to flicker light induced provocation, maximal artery dilation was greater in the black group than in the white group [46]. 

A study made on small children, aged between 4 and 5 years, showed wider CRAE and CRVE. The CRAE values were 180.9 ± 14.2 µm and the CRVE values were 251 ± 19.7 µm [47]. Moreover, black South African children presented wider retinal venules than white South African children [48].

## 4. Retinal Vascular Changes in Cardiovascular Disease

**Figure 2:**
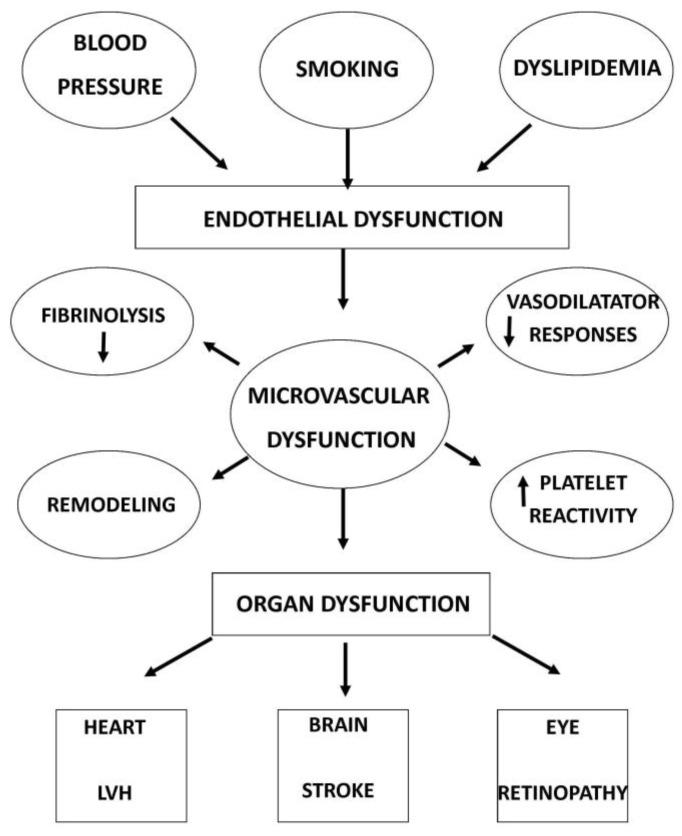
Causes and consequences of microvascular dysfunction.

Studies have analysed the close link between retinal microvascular changes and systemic pathologies, such as cardiovascular risk [49] and cardiovascular mortality [43]. The research of ocular biomarkers for studying systemic disease is now conceptualized as “oculomics” [50] (Figure 2).

### 4.1. Retinal Vascular Changes and Heart Disease

Retinal vessel analysis has been used to evaluate cardiovascular diseases for a long time. Different population-based studies showed an important link between the retinal vasculature parameters and cardiovascular risk in older populations [51,52]. Atherosclerosis is known as the most important cause of cardiovascular disease. It is characterized by chronic inflammation of the blood vessels. Non-invasive analysis of retinal microvasculature can reveal significant dysfunction of vessels, and has the potential to predict cardiovascular events in the general population [53]. Fu Y et al. performed a study on 57,947 participants without a history of CVD that were followed for a period of 11 years. In total, 3211 cardiovascular events occurred during the follow-up. The authors found decreasing fractal dimensions (adjusted HR = 0.80, 95% CI, 0.65–0.98, *p* = 0.033), lower number of vascular segments of arteries (adjusted HR = 0.69, 95% CI, 0.54–0.88, *p* = 0.002) and venules (adjusted HR = 0.77, 95% CI, 0.61–0.97, *p* = 0.024). Reduced arterial vascular skeleton density (adjusted HR = 0.72, 95% CI, 0.57–0.91, *p* = 0.007) and venous vascular skeleton density (adjusted HR = 0.78, 95% CI, 0.62–0.98, *p* = 0.034) were associated with an increased risk of cardiovascular pathologies [53]. Retinal arteriolar endothelial dysfunction correlates with the severity of cardiovascular diseases and can be used as a predictor for major cardiovascular events [54]. Al-Fiadh et al. performed a prospective study on 197 subjects. In order to assess retinal microvascular endothelial dysfunction, the authors measured retinal arteriolar and venular dilatation to flicker light, expressed as a percentage increase over the baseline diameter. They showed that, in patients with coronary artery disease, the mean retinal arteriolar dilatation was attenuated compared with controls. After adjustment for cardiovascular risk factors and age, retinal arteriolar dilatation was independently correlated with coronary artery disease [55].

A meta-analysis showed that a lower CRAE value might be associated in women with a higher risk of coronary heart disease [56]. Schuster et al. analysed several cardiovascular parameters in a working age population. CRAE/CRVE and AVR were analysed using validated software. They found that smaller CRAE was associated with increased risk of higher arterial blood pressure, higher age and higher body mass index. CRVE was inversely associated with age. AVR showed a significant association to arterial blood pressure and body mass-index [57]. The authors revealed that the lower density of the retinal vascular network may correlate with an increased cardiovascular risk. They suggest that a snap shot of the retinal vessels may indicate the relative risk for cardiovascular events.

The relation between retinal vascular geometry and cardiovascular disease has been reported by the Australian Heart Eye Study [58]. This is a cross-sectional study that included 1680 patients that underwent coronary angiography for the evaluation of potential coronary artery disease. They obtained a range of quantified retinal vessel geometric measurements using retinal photographs. The authors found a link between straighter retinal arterioles and venules and the severity of coronary artery disease [58]. They also reported that lower fractal dimensions, indicating a sparser retinal microvascular network, are associated with the severity of coronary artery disease and with greater risk of atrial fibrillation. In the Rotterdam study, a 25 year follow-up study, the authors found that retinal vessel diameters correlated with long-term survival rate. Arteriolar narrowing and venular widening were associated with higher risk of cardiovascular mortality [59].

Shokr et al. conducted a study on 123 participants with low cardiovascular risk, aimed to assess the role of retinal vascular function as a predictor for systemic blood pressure. They study included two groups, one with younger participants and another with older ones. The authors identified age-related differences between the study groups in retinal arterial time to maximum dilation, maximum constriction and maximum constriction percentage. In the youngest participants, the error between predicted versus actual values for the chronological age was smallest in cases of using both retinal vascular functions only, or the combination of this parameter with the relative telomere length. Their results showed a better correlation between retinal vascular function, telomere length and chronological age in individuals under 30 years of age. Systolic blood pressure was better predicted by telomere measurements [60].

### 4.2. Retinal Vessel Analysis in Adults with Hypertension

Several studies showed the link between retinal arteriolar narrowing and the occurrence of arterial hypertension [61,62]. Table 5 presents the vascular changes in arterial hypertension (Table 5). The Blue Mountain Eye Study found a significant correlation between arteriolar narrowing and hypertension severity [62]. In the Multi-Ethnic Study of Atherosclerosis (MESA), the authors confirmed the importance of arteriolar narrowing as a prognostic factor and also found an association between the retinal venular widening and the development of hypertension [20].

In a meta-analysis, Chew et al. confirmed that arteriolar narrowing indicates an increased risk for hypertension [63]. It was estimated that for each 10 mmHg increase in mean blood pressure, the retinal arteriolar diameter reduces by about 3 μm. In the Gutenberg Health Study, the authors identified narrower retinal arterioles in participants with untreated hypertension [44]. In one study that included 189 patients, within a 6 month treatment program, 74% achieved a blood pressure in the normal range, which was associated with wider retinal arterioles and a higher AVR value [64].

Alterations in retinal vascular bed might be associated with subclinical left ventricular systolic and diastolic dysfunction [65]. In their study, Chandra et al. showed that decreased CRAE and increased CRVE values were associated with echocardiographic measures of both left ventricular systolic and diastolic dysfunction [65]. In another study, decreased retinal venular branching angle and fractal dimension were independently associated with left ventricular and left atrial dysfunction [66].

### 4.3. Retinal Vascular Changes and CVD Mortality

Changes in retinal vessels correlate with a high risk of CVD mortality [67]. We present the retinal changes in heart failure and stroke (Table 6). In the Blue Mountains Eye Study, patients with signs of retinopathy had a greater risk of coronary heart disease [68]. In the National Health and Nutrition Examination Survey (NHANES), the risk of CVD mortality in patients with both retinopathy and chronic kidney disease, was increased more than 2-fold, as compared to patients with neither retinopathy nor chronic kidney disease [69]. Sairenchi et al. showed in their study that both hypertensive and non-hypertensive participants with signs of mild retinopathy had a greater risk of dying from CVD [70].

## 5. Exercise Improves Retinal Microvascular Health

Exercise has an important role, as it counteract microvascular remodelling and decreases the risk of small vessel disease [5]. Physical activity and exercise play a key role in the prevention of CVD, while smoking, a high calorie diet and a sedentary lifestyle increase the risk of CV pathologies. Exercise protects against endothelial dysfunction, playing an important role in the prevention of CVD [73]. In a cohort study on more than one million patients, the authors showed that moderate physical activity determined a decrease in cardiovascular risk of 11%. An increase in sedentarism resulted in an increase in cardiovascular risk by 27% [74]. Physical activity was associated with narrower CRVE and higher AVR, while physical inactivity was associated with narrower CRAE and wider CRVE [75]. In healthy older adults, physical activity was associated with higher AVR and wider CRAE, compared to healthy older sedentary adults [76]. A study by Hanssen et al. showed that obese runners benefited the most from high intensity training, as compared to healthy athletes, with wider CRAE and higher AVR after 10 weeks of training [77]. In another study, healthy sedentary individuals showed higher flicker-light induced retinal vessel dilatation (FID) compared to healthy active individuals [78]. The EXAMIN AGE study examined the effects of exercise on retinal FID. The authors found an improvement in aFID in patients with CV risk and high-intensity interval training when compared to controls [79].

## 6. Artificial Intelligence in Retinal Vessel Analysis

Currently, retinal vascular changes are manually or semi-automatedly assessed, following standardized protocols. The semi-automated analysis demonstrated a link between morphological changes in the retinal vasculature and systematic pathologies. Researchers thought to use artificial intelligence (AI) techniques to improve analysis. Fundus processing using these techniques may help investigators to easily detect retinal biomarkers for cardiovascular risk.

The term AI was used in 1955 by John McCarthy to describe computer systems capable of performing complex tasks that only humans can do [80]. AI is useful for description tasks, such as finding relationships within a dataset without a defined outcome. It uses computer algorithms to learn from raw data and to create a representation of this data [81]. The receiver operating characteristics area under curve (AUC) is used to evaluate machine learning algorithms against a “gold standard” of either human or objective diagnostic measures [82].

Recent AI developments in medicine promises an improvement in screening and diagnostics of different pathologies [83]. Retinal vascular imaging using AI may help identify the cardiovascular risk. Researchers considered different automatic analysis algorithms in order to identify markers of retinal vascular health. These markers may be used to confirm the link between retinal microvasculature changes and cardiovascular status [84]. Poplin et al. have shown that specific cardiovascular risk factors—age, gender, blood pressure, body mass index, smoking and HbA1c, can be predicted using deep learning (DL). Using DL models trained on data from 284,335 participants and validated on two independent databases, the authors predicted major cardiac events with an AUC of 0.70 [19]. 

Machine learning (ML) and DL have an important potential for quantification of retinal vascular biomarkers [85,86]. The performance of automated retinal diseases classification by DL systems was shown to be superior to that of human specialists [18].

ML is used to build clinical decision systems. It is a subset of AI that creates programmes based on large datasets [87]. DL is another subset of AI. Its aim is to copy the structure of the central nervous system by creating artificial neural networks using convolutional neural networks (CNNs). These networks, which are of high interest for the field of retinal imaging, are trained with large, annotated datasets, allowing computers to recognize visual patterns [88].

Different researchers used high quality retinal imaging databases, such as MESSIDOR, STARE project, DRIVE, E-ophtha and EyePACS [89,90]. The most commonly used retinal images were fundus photographs, OCT and OCTA images.

Software that uses automated image processing is QUARTZ. Quantitative Analysis of Retinal vessel Topology and size (QUARTZ) distinguishes between venules and arterioles. Moreover, it identifies vessel segmentation, measures vessel width and angular changes and tortuosity [91,92]. Cheung et al. developed a DL algorithm that uses retinal photographs in order to automatically measure the retinal vessel calibre (SIVA-DLS) [93]. Based on more than 20,000 fundus images, Kim et al. showed an accurate prediction for age with the CNN ResNet-152 algorithm [94]. They found that, in patients with hypertension and diabetes mellitus, the differences between the predicted age and the chronological age were higher after the age of 60. 

Arnould et al. focused on quantitative ‘’oculomics” obtained from the Singapore “I” Vessel Assessment (SIVA) [95]. They used algorithms based on combined retinal fundus images and OCTA vascular metrics to predict age, diabetes mellitus and hypertension.

The coronary artery calcium (CAC) score was developed to better stratify patients with cardiovascular risk. This score is a biomarker of atherosclerosis, and it is calculated from cardiac CT measurements [96]. Since these measurements are invasive, Son et al. presented a DL algorithm that helps differentiate between patients with high CAC scores and patients with low CAC scores, based on retinal fundus photographs [97]. They demonstrated a moderate AUC of 0.832 with bilateral fundus images. SIVA-deep learning system (DLS) automatically measures the retinal vessel calibre, using retinal photographs [93]. It uses CNN to estimate CRAE and CRVE within 0.5–2 disc diameters away from the optic disc. It measures the CRAE, CRVE values from SIVA. The retinal arteriolar calibre measured with SIVA-DLS offers a significant prediction rate for cardiovascular events [93]. The authors demonstrated that narrower CRAE was associated with CVD events. This method is straightforward, but might lack interpretability and has limited output parameters. Nusinovici et al. developed a DL algorithm to predict the likelihood of being over 65 years old, by using retinal fundus images (“RetiAGE”) [98]. The RetiAGE used biomarkers such as age and glucose, albumin, C-reactive protein, creatinine, lymphocytes, red cell volume, white blood cells to predict cardiovascular mortality. They reported a significant prediction rate for cardiovascular mortality with an HR of 2.42 (95% CI 1.69–3.48).

Advances in the field of AI improved of OCTA images analysis. Ma et al. presented the Retinal OCTA Segmentation dataset (ROSE), which helps with vessels’ segmentation using OCTA images [99]. Recently, other retinal biomarkers have been introduced (automated foveal avascular zone measurement, retinal vessel calibre and tortuosity measurements) to help identify the cardiovascular risk [100,101,102].

Shi D aimed to validate a new AI system (Retina-based Microvascular Health Assessment System, RMHAS) for automated vessel segmentation. RMHAS addresses the limitations of existing algorithms and software—IVAN, SIVA and VAMPIRE. Even if the QUARTZ platform can analyse whole fundus images, it does not have many output parameters. The RMHAS algorithm provides more physical and geometric parameters. In addition to standard vessel calibre measurements, it offers measurements on tortuosity and additional topological information [103]. The ORAiCLE and THEIA systems (Toku Eyes, Auckland, New Zealand) are undergoing FDA approval. They aim to use AI to help detect cardiovascular and renal risk factors by analysing retinal funduscopic images.

The introduction of AI into clinical practice has the advantage of increasing diagnosis accuracy and improving patient care by complementing the role of physicians. On the other hand, AI faces some challenges such as data reliability, medicolegal issues, and alteration of the patients-physician relationship [104].

## 7. Clinical Implementation in Cardiovascular Disease Prevention

Individuals with low and intermediate CV risk have the biggest advantage in using these vascular biomarkers. If one of the vascular markers is abnormal, the risk of developing the disease is higher and the need for aggressive treatment is evident.

As mentioned above, consistent studies demonstrated the link between retinal vascular changes and CV risk (Table 7). Recent data from the ARIC study, which included 10,470 asymptomatic adults followed over a period of 16 years, show that narrower retinal arterioles and wider venules can be linked to a greater risk of CVD events in women [105]. Retinal vessel diameters and retinal FID are the most important biomarkers to improve CV risk prediction [106]. A prospective study that included diabetic patients showed that using retinal vessel analysis adds significant value to reclassifying CVD risk [107]. Hypertension treatment is associated with significant CVD risk reduction. Studies showed that blood pressure reduction correlates with the regression of retinal vascular changes [64,108].

## 8. Conclusions

The important link between retinal vessel diameters and cardiovascular risk factors is already a well-known fact. The study of the retinal vascular bed helps identifying microcirculation changes in cardiovascular diseases. Advanced retinal vascular imaging technologies have been developed to allow a more precise assessment of retinal vascular changes. The introduction of AI into clinical practice has the advantage of increasing diagnosis accuracy and improving patient care by complementing the role of physicians. On the other hand, AI faces some challenges such as data reliability, medicolegal issues, and alteration of the patient-physician relationship.

New emerging data on their clinical utility show the importance of retinal vessel diameters and flicker light-induced dilation as candidate microvascular biomarkers in predicting cardiovascular events. Physical activity and exercise are associated with a favourable retinal microvascular phenotype. In patients with cardiovascular risk, physical exercise can counteract endothelial dysfunction. Retinal vessel analysis is an important tool in primary CV disease prevention, as it can identify individuals at risk and can determine the initiation of early treatment strategies. This suggests that retinal vasculature changes might indicate early alterations within the microvasculature before cardiovascular diseases occur.

Further research should explore the potential clinical application of retinal microvascular biomarkers, in order to assess systemic vascular health status, and to predict cardiovascular events.

## Figures and Tables

**Table 1:** Overview of retinal vessel analysis methods [17].

Method	Main Content	Principle of Measurement
Retinal Vessel Analyzer	Vessel diameter	Fundus camera-based imaging technology
Laser Doppler Velocimetry	Blood flow velocity	Optical Doppler shift
Dye-based Angiography	Visualization of anatomic structures	Passage of a fluorescent dye
OCTA	Depth resolved angiograms	Detection of intravascular moving particles as an intrinsic contrast
Doppler OCTA	Retinal blood flow	Generation of a reflective profile and detection of phase shifts of the back-scattered lights
Retinal oximetry	Oxygen saturation of haemoglobin in RBCs	Difference in absorption of light between oxyhaemoglobin and deoxyhaemoglobin
Laser Speckle flowgraphy	Mean Blur Rate (MBR) as relative index of retinal blood flow velocity	Detection of changes in the speckle pattern by reflection of coherent laser light

**Table 2:** Retinal vascular changes measured on retinal photographs.

	Vascular Changes	Description
Retinopathy signs	Haemorrhages	red deposits—exudation of blood
	Exudates	yellow deposits—exudation of lipids
	Cotton-wool spots	fluffy white lesions—ischemia of nerve fibre layer
Retinal arteriolar wall signs	Focal arteriolar narrowing (FAN)	constriction of retinal arterioles
	Arteriovenous nicking/ nipping (AVN)	arteriovenous crossing
	Opacification of arteriolar wall (OAW)	Silver wiring of retinal arterioles
Quantitative retinal vascular parameters	Central retinal artery equivalent (CRAE)	index reflecting the average width of retinal arterioles
	Central retinal venule equivalent (CRVE)	index reflecting the average width of retinal venules
	Tortuosity	a measure of the curliness of the retinal vessels
	Fractal dimension	branching complexity of the capillary network
	Branching angle	first angle subtended between two daughter vessels at each vascular bifurcation
	Branching coefficient	ratio of the branching vessel widths to trunk vessel width
	Length-diameter-ratio	ratio of the length between 2 branching points to trunk vessel width

**Table 3:** Reference values of AVR, CRAE and CRVE [44].

Reference Values (2.5th–97.5th Percentile)	Men		Women	
	<55 Years	>55 Years	<55 Years	>55 Years
AVR	0.72–0.98	0.72–0.95	0.76–0.99	0.73–1.01
CRAE	143.07–213.74	129.15–202.49	155.54–220.58	145.02–217.07
CRVE	179.03–242.69	170.52–241.99	182.32–245.98	178.21–259.72

**Table 4:** Association of the AVR, CRAE and CRVE with cardiovascular risk factors and diseases [44].

Risk Factors and Diseases	AVR			CRAE			CRV		
	OR	95% CI	*p*	OR	95% CI	*p*	OR	95% CI	*p*
Age	0.985	0.974–0.996	0.010	0.965	0.951–0.980	0.01	0.981	0.965–0.998	0.025
Hypertension	2.703	2.098–3.848	0.001	2.881	2.099–3.954	0.001	0.786	0.549–1.127	0.190
Diabetes	0.564	0.335–0.948	0.031	0.405	0.169–0.976	0.044	0.649	0.250–1.686	0.374
Smoking	0.843	0.628–1.131	0.255	0.653	0.437–0.977	0.038	0.555	0.341–0.902	0.018
Dyslipidaemia	1.073	0.842–1.367	0.568	1.189	0.864–1.638	0.288	0.797	0.540–1.178	0.255
Obesity	1.031	0.798–1.331	0.818	0.813	0.571–1.158	0.251	0.727	0.470–1.125	0.153
Heart failure	0.465	0.142–1.518	0.204	1.222	0.368–4.054	0.743	1.317	0.356–4.878	0.680
Stroke	1.308	0.610–2.803	0.491	1.178	0.371–3.740	0.781	1.100	0.299–4.054	0.886
Myocardial infarction	1.326	0.684–2.572	0.403	0.403	0.090–1.798	0.234	1.711	0.568–5.158	0.340

**Table 5:** Vascular changes in arterial hypertension.

AV Crossing Changes	Arterial Changes	Retinal Changes	Macular Changes	Optic Nerve Changes
Salus’s sign—deflection of retinal vein as it crosses the arteriole	Decrease in the AV ratio to 1:3	Retinal haemorrhages - Dot-blot haemorrhages - Flame shaped haemorrhages	Macular star formation due to depositing of hard exudates around the macula	Optic disk swelling
Gunn’s sign—tapering of the retinal vein on either side of the AV crossing	Changes in the arteriolar light reflex—copper or silver wiring	Retinal exudates - Hard exudates—lipid deposits in the retina - Soft exudates—cotton wool spots due to ischemia		
Bonnet’s sign—banking of the retinal vein distal to the AV crossing				

**Table 6:** Retinal microvascular alterations in patients with heart failure and stroke [71,72].

Microvascular Alterations in Heart failure	Microvascular Alterations in Stroke
Wider retinal venular calibre Lower vascular fractal dimension Smaller number of vessel segments	Retinal arteriole narrowing Retinal arteriovenous nicking Lower fractal dimension Haemorrhage Microaneurysm

**Table 7:** Studies that show the link between retinal vascular changes and CV diseases.

Study	Country	Retinal Vessel Analysis Software	Retinal Vascular Changes	References
McGeechan 2008	USA		Arteriolar narrowing, venular widening and arteriolar walls signs—associated with incident coronary heart disease	[56]
Myers 2012	USA		Arteriolar narrowing and venular widening were associated with coronary heart disease and stroke mortality	[52]
Liew 2009	Australia		Arteriolar narrowing and venular widening associated with coronary heart disease and stroke mortality	[68]
Kawasaki 2009	USA		Arteriolar narrowing, retinopathy associated with incident stroke and stroke mortality	[20]
Ricardo 2014	USA		Retinopathy—cardiovascular mortality in persons with chronic kidney disease	[69]
Mutlu 2016	The Netherlands		Venular widening—incident stroke	[59]
Siantar 2015	Singapore		Venular widening, arteriolar narrowing, retinopathy—incident CVD in diabetics	[67]
Patel 2022	United Kingdom	DVA	Reduced flow-mediated dilation responses associated with a reduced baseline-corrected microvascular arterial dilation response to flickering light	[54]
Köchli 2022	South Africa, Switzerland	RVA	Narrower CRAE associated with higher body mass index and blood pressure BP	[48]
Hanssen 2022	Switzerland	RVA, DVA	Narrower CRAE and higher arteriolar flicker induced dilation associated with higher blood pressure	[8]
Theuerle 2021	Australia	DVA	Lower flicker light-induced retinal arteriolar dilation associated with higher risk of major adverse cardiovascular events	[106]
Cheung 2021	Multicountry	SIVA-DLS	Associations between measurements of retinal-vessel calibre and CVD risk factors, including BP, body mass index, total cholesterol and glycated-haemoglobin levels	[93]
Poplin 2018				[19]
Wei 2019	Belgium	IVAN	Smaller CRAE is associated with higher central pulse pressure, pulse wave velocity	[26]
Ponto 2017	Germany	RVA	Lower CRAE and AVR in participants with uncontrolled hypertension	[44]
Chandra 2019	USA	ARIC protocol for RVA (Hubbard)	CRVE widening and CRAE narrowing were associated with larger left ventricular size, higher prevalence of left ventricular hypertrophy	[65]
Tapp 2019	United Kingdom	QUARTZ	Narrower arterioles were associated with higher systolic BP, higher mean arterial pressure. Greater arteriolar tortuosity was associated with higher systolic BP, higher mean arterial pressure and higher pulse pressure	[92]
Madhloum 2020	Belgium	MONA	Reference values for CRAE and CRVE	[47]
Shokr 2020	United Kingdom	DVA	Microvascular alterations can be identifiable at normal values BP, associated with changes in oxidative stress level	[60]
Takayama 2018	Japan	RVA	Correlations between age, intraocular pressure, axial length, and choriocapillaris vasculature	[42]
Cífková 2021	Czech Republic	laser Doppler flowmetry (SLDF)	Juxtapupillary retinal capillary blood flow increased with age, while vessel and luminal diameters decreased. Systolic blood pressure correlates with wall thickness	[45]
Smith 2020	African countries	RVA	Black participants had a smaller CRAE value than white participants. In response to flicker light induced provocation, maximal artery dilation was greater in the black than in the white group	[46]

## Data Availability

Data sharing not applicable.
